# Targeting endoplasmic reticulum stress in diabetic retinopathy: mechanistic insights and emerging therapies

**DOI:** 10.1186/s40659-026-00675-0

**Published:** 2026-01-28

**Authors:** Junting Weng, Rongjie Guo, Danjuan Liu, Shanjiao Huang, Shuoyun Weng

**Affiliations:** 1 Department of Critical Care Medicine, The Affiliated Hospital of Putian University, Putian, 351100 China; 2 School of Ophthalmology & Optometry, Wenzhou Medical University, No. 270 Xueyuan Road, Lucheng District, Wenzhou City, 325000 Zhejiang Province China

**Keywords:** Diabetic retinopathy, Endoplasmic reticulum stress, Inflammation, Oxidative stress, Apoptosis

## Abstract

**Purpose:**

To summarize the role of endoplasmic reticulum stress (ERS) in the pathogenesis of diabetic retinopathy (DR) and evaluate potential ERS-targeted interventions.

**Methods:**

This review analyzes recent preclinical and clinical studies focusing on the molecular mechanisms of ERS and its impact on retinal inflammation, oxidative stress, and angiogenesis in DR.

**Results:**

ERS, triggered by hyperglycemia-induced oxidative stress and glucotoxicity, activates the unfolded protein response (UPR) via inositol-requiring enzyme 1 (IRE1), PKR-like endoplasmic reticulum kinase (PERK), and activating transcription factor 6 (ATF6) pathways. While initially protective, prolonged ERS leads to apoptosis, chronic inflammation, and neovascularization. Key downstream mediators include C/EBP homologous protein (CHOP), X-box binding protein 1 (XBP1), and activating transcription factor 4 (ATF4). ERS inhibitors such as 4-phenylbutyric acid and tauroursodeoxycholic acid, along with selective modulators of UPR signaling, have shown neuroprotective and anti-inflammatory effects in DR models. Combination therapies integrating antioxidants and anti-inflammatory agents demonstrate synergistic efficacy. However, clinical translation remains limited by delivery barriers and incomplete understanding of UPR-specific actions in the human retina.

**Conclusion:**

Targeting ERS presents a promising therapeutic strategy for DR, with the potential to preserve vision and improve outcomes for diabetic patients. Future research should focus on elucidating the precise molecular pathways and developing targeted, personalized ERS-modulating therapies.

## Introduction

Diabetic retinopathy (DR) is a leading cause of vision loss and one of the most severe microvascular complications associated with diabetes mellitus [1]. The disease progresses through multiple stages, commencing with mild non-proliferative diabetic retinopathy (NPDR), which is characterized by microaneurysms, capillary leakage, and vascular endothelial dysfunction. As DR advances, it transitions into moderate and severe NPDR, where vascular occlusion, hemorrhages, and increased capillary permeability contribute to progressive deterioration of retinal function. The final stage, proliferative diabetic retinopathy (PDR), is marked by pathological neovascularization, leading to fibrosis, vitreous hemorrhage, and retinal detachment, which can ultimately result in irreversible vision loss [2]. Additionally, diabetic macular edema (DME) may develop at any stage, further exacerbating visual impairment through fluid accumulation in the macula [3].

The pathophysiology of DR is primarily driven by chronic hyperglycemia, which induces oxidative stress, inflammation, mitochondrial dysfunction, and endothelial damage [4]. Hyperglycemia activates several metabolic pathways, including the polyol pathway, protein kinase C (PKC) signaling, and advanced glycation end-product (AGE) formation, all of which contribute to retinal vascular dysfunction. Inflammatory mediators such as tumor necrosis factor-alpha (TNF-α), interleukin-6 (IL-6), and nuclear factor-kappa B (NF-κB) play a critical role in promoting endothelial cell apoptosis, pericyte loss, and blood-retinal barrier (BRB) breakdown. Oxidative stress, predominantly caused by excessive reactive oxygen species (ROS) production, further exacerbates retinal damage and promotes microvascular occlusion [5].

Recent research has underscored the pivotal role of endoplasmic reticulum stress (ERS) in the pathogenesis of DR [6]. Under hyperglycemic conditions, prolonged ERS disrupts retinal homeostasis, resulting in the accumulation of misfolded proteins and activation of the unfolded protein response (UPR) [7]. The three major UPR pathways—Inositol-Requiring Enzyme 1 (IRE1), PKR-Like Endoplasmic Reticulum Kinase (PERK), and Activating Transcription Factor 6 (ATF6)-are involved in cellular adaptation to ER stress [8]. Although transient UPR activation serves a protective role by restoring protein-folding homeostasis, persistent ERS triggers inflammatory responses, oxidative stress, and apoptosis, which can contribute to retinal neurodegeneration and microvascular dysfunction [9, 10].

Although the roles of ERS in diabetes-related complications have been reviewed elsewhere, our review discussed this topic from different perspectives. Oshitari et al. [11] provided insights into the molecular mechanisms of ERS in DR, but they did not address emerging therapeutic strategies or the interplay between UPR pathways and inflammation. Cameron [12] emphasized ERS in diabetic neuropathy, however, the role of ERS in retinal-specific pathologies remained unexplored. Alam et al. [13] highlighted ERS in retinal cells, but they did not discuss it from the aspects of preclinical and clinical advancements, including gene-editing technologies and combination therapies. To our knowledge, none of these reviews comprehensively and critically discussed translational potential, UPR pathway crosstalk in DR progression, and anti-inflammatory/antioxidant synergies with ERS modulation. Herein, we provide an up-to-date review by (1) thoroughly analyzing the molecular mechanisms of the three major UPR pathways-IRE1, PERK, and ATF6-in the context of DR; (2) comprehensively examining the interplay among ERS, inflammation, oxidative stress, apoptosis, and aberrant angiogenesis; and (3) highlighting recent progress in therapeutic interventions, including ERS inhibitors, UPR pathway modulators, antioxidants, anti-inflammatory agents, and gene-editing technologies. By offering an integrated and forward-looking analysis, this review can provide novel insights into translational research of ERS for the effective treatment of DR.

## Epidemiology of DR

DR affects approximately 35% of individuals with diabetes globally, with disease severity closely associated with poor glycemic control and genetic predisposition [14]. Key risk factors include prolonged hyperglycemia (HbA1c > 7%), hypertension, and dyslipidemia, all of which exacerbate ERS through oxidative and inflammatory cascades [15]. Notably, racial disparities in DR prevalence-such as higher rates observed in African and South Asian populations-may be attributed to genetic variants in vascular endothelial growth factor (VEGF) and UPR-related genes [16]. These findings highlight ERS as a modifiable therapeutic target in high-risk populations.

## Pathophysiology of DR

The pathophysiological process of DR is characterized by complex pathological changes driven by a hyperglycemic microenvironment, encompassing microvascular dysfunction, chronic inflammation, oxidative stress, neurodegeneration, and abnormal angiogenesis [17]. Hyperglycemia induces oxidative stress and endothelial cell dysfunction via metabolic pathways such as the polyol pathway, PKC signaling, and advanced glycation end products (AGEs), leading to BRB disruption, endothelial cell and pericyte loss, capillary occlusion, and microaneurysm formation [18]. Retinal hypoxia secondary to vascular occlusion further activates hypoxia-inducible factor-1α (HIF-1α) and VEGF, promoting pathological neovascularization-a hallmark feature of proliferative diabetic retinopathy (PDR) [19]. Concurrently, hyperglycemia induces chronic low-grade inflammation, activating NF-κB and NLRP3 inflammasome pathways, thereby promoting the release of pro-inflammatory cytokines (e.g., TNF-α, IL-6) and exacerbating retinal damage. This inflammatory cascade is accompanied by increased leukocyte adhesion, endothelial cell apoptosis, and enhanced vascular permeability [20]. Oxidative stress constitutes a critical pathological mechanism of DR, wherein high glucose levels induce intracellular oxidative damage through increased ROS production and suppression of antioxidant enzyme activity, subsequently activating ERS and mitochondrial apoptosis pathways [21]. Retinal neurodegeneration emerges as an important early feature of DR, with hyperglycemia inducing ganglion cell apoptosis, glial cell activation, and disruption of neuron-vascular coupling, ultimately impairing visual function [22]. In the PDR stage, abnormal neovascularization, coupled with fibrous tissue proliferation, may culminate in tractional retinal detachment, resulting in irreversible vision loss [23]. These pathological mechanisms are intricately interconnected, forming a vicious cycle of inflammation, oxidative stress, ER stress, and microvascular dysfunction, which serves as the primary driving force behind DR progression.

## Molecular basis of ER stress

ER is a critical organelle responsible for protein folding, post-translational modification, calcium homeostasis maintenance, and lipid synthesis in eukaryotic cells. Under normal physiological conditions, the ER ensures that synthesized proteins are properly folded and transported to their respective destinations within the cell via its efficient folding machinery [24]. However, when cells are exposed to adverse conditions such as hyperglycemia, hypoxia, or oxidative stress, the protein-folding function of the ER may be disrupted, leading to the accumulation of unfolded or misfolded proteins and the activation of ERS [25]. In response to this stressed state, cells activate the UPR, which aims to restore cellular homeostasis. If the stress persists or becomes excessive, however, the UPR may transition into a pro-apoptotic signaling pathway, ultimately resulting in cell death [25]. The UPR serves as the central regulatory mechanism of ERS, coordinating protein folding, degradation, and cellular stress responses through the activation of three major sensor proteins: IRE1, PERK, and ATF6. These pathways work synergistically to restore ER homeostasis but may also induce apoptosis under sustained stress conditions [26].

### IRE1 pathway

IRE1 is one of the core sensors of ERS and represents the most evolutionarily conserved signaling pathway within the UPR. As a bifunctional enzyme with both kinase and endonuclease activities in its intracellular domain, IRE1 initiates a variety of downstream signals upon detecting the accumulation of unfolded proteins in the ER, thereby regulating cell fate [27].

#### Structure and activation mechanism of IRE1

IRE1 is an ER-resident transmembrane protein containing a luminal stress-sensing domain and a cytosolic kinase/endoribonuclease domain. When unfolded or misfolded proteins accumulate in the ER, these proteins bind to the extracellular domain of IRE1, inducing its dimerization or oligomerization [28]. This dimerization triggers autophosphorylation of the intracellular kinase region and activates the endonuclease activity. Activated IRE1 specifically cleaves X-box binding protein 1 (XBP1) mRNA through its endonuclease function, generating the active spliced form XBP1s. XBP1s functions as a transcription factor that translocates to the nucleus and induces the expression of genes involved in protein folding and degradation [29].

#### Biological function of IRE1 pathway

##### Promote protein folding and degradation

XBP1s-induced gene expression includes molecular chaperones such as GRP78 and components of the endoplasmic reticulum-associated degradation (ERAD) system. These proteins enhance the ER’s capacity to process misfolded proteins, alleviate stress, and maintain ER homeostasis [30].

##### Regulation of inflammatory response

IRE1 activates the c-Jun N-terminal kinase (JNK) and NF-κB signaling pathways through its kinase activity, which in turn can induce the expression of pro-inflammatory cytokines. Inflammatory responses play a significant role in chronic diseases such as DR, where inflammation drives retinal microvascular dysfunction and neurodegeneration [31].

##### Induction of apoptosis

Sustained activation of IRE1 induces mitochondrial apoptotic pathways via JNK signaling. JNK promotes the translocation of pro-apoptotic factors such as Bax and inhibits anti-apoptotic factors such as Bcl-2, leading to the loss of mitochondrial membrane potential and apoptosis. Additionally, overactivation of IRE1 enhances the expression of C/EBP homologous protein (CHOP), further exacerbating cell death [32, 33].

##### Regulation of metabolism and oxidative stress

The IRE1 pathway plays a key role in regulating cellular metabolism. For example, by modulating genes involved in lipid metabolism, IRE1 helps mitigate ERS that is caused by hyperlipidemia. Excessive activation of IRE1, however, intensifies ROS production, forming a positive feedback loop between oxidative stress and ERS, thereby further damaging retinal cells [34].

### PERK pathway

PERK is another core sensor of ERS and plays a pivotal role in regulating the UPR. As a transmembrane kinase, PERK alleviates stress and restores homeostasis in cells by inhibiting global protein translation and regulating gene expression upon detecting the accumulation of unfolded or misfolded proteins in the ER. However, when stress persists in the cell, the PERK pathway may shift from a protective response to a pro-apoptotic signal, contributing to cell damage and disease progression [35].

#### PERK activation mechanism and signal transduction

PERK resides in the endoplasmic reticulum membrane. Upon the accumulation of unfolded proteins, the N-terminal of PERK dissociates from the molecular chaperone GRP78 (BiP), exposing its activation site. PERK activates its C-terminal kinase domain through dimerization and autophosphorylation, initiating downstream signaling. Activated PERK inhibits global protein translation by phosphorylating eukaryotic translation initiation factor 2α (eIF2α). This process reduces the burden of nascent proteins within the ER while allowing selective translation of stress-related proteins such as activating transcription factor 4 (ATF4). Phosphorylated eIF2α promotes the translation of ATF4, which translocates to the nucleus and regulates the expression of multiple stress-related genes, including antioxidant proteins, amino acid transporters, and molecular chaperones, helping cells respond to ERS [36]. However, under excessive stress, ATF4 induces the expression of the pro-apoptotic gene CHOP. CHOP is a key downstream effector of the PERK pathway, activating the mitochondrial apoptosis pathway by downregulating anti-apoptotic proteins such as Bcl-2 and upregulating pro-apoptotic factors such as Bax and Bim. Additionally, CHOP exacerbates cell damage by inducing oxidative stress [37].

#### Biological function of PERK pathway

The PERK pathway mitigates ERS in the short term by inhibiting protein synthesis and enhancing antioxidant capacity, which is essential for maintaining ER homeostasis [38]. Continuously activated PERK promotes cell death through CHOP and mitochondrial apoptosis pathways. This pro-apoptotic function eliminates damaged cells when ER stress exceeds the cell’s coping ability but can lead to tissue damage in chronic diseases. ATF4 regulates antioxidant genes such as heme oxygenase-1 (HO-1), enhancing the cell’s ability to clear ROS [39]. However, the upregulation of CHOP creates a positive feedback loop, exacerbating ROS production and leading to cellular oxidative damage [40].

### ATF6 pathway

ATF6 is one of the three major signaling pathways involved in the UPR. Unlike IRE1 and PERK, ATF6 is an ER-resident transmembrane transcription factor that, upon ER stress, translocates to the Golgi where it is cleaved by S1P/S2P proteases. The released N-terminal fragment enters the nucleus to regulate genes involved in ER protein folding, ER-associated degradation (ERAD), and stress adaptation [41].

#### Activation mechanism and signal transduction of ATF6

The N-terminal of ATF6 resides in the ER. When unfolded or misfolded proteins accumulate in the ER, the chaperone GRP78 (BiP) binds to the unfolded proteins and dissociates from ATF6. As a consequence, ATF6 is transported to the Golgi apparatus, where it is cleaved by two proteases, S1P (site 1 protease) and S2P (site 2 protease), releasing its intracellular soluble fragment (ATF6f). The cleaved ATF6f then migrates to the nucleus, binds to the promoter regions of UPR target genes, and activates the expression of molecular chaperones (e.g., GRP78 and GRP94), folding enzymes (e.g., PDI), and genes associated with the ERAD system [42]. Eventually, the expression of these genes enhances the cell’s ability to respond to ERS. ATF6 not only promotes cell survival by inducing adaptive gene expression but also participates in the regulation of cell death through cross-activation of CHOP or other pro-apoptotic factors during excessive stress [43].

#### Biological function of ATF6 pathway

##### Enhanced protein folding and endoplasmic reticulum function

ATF6 augments the protein-folding capacity of the ER through the transcriptional upregulation of molecular chaperones (e.g., GRP78) and folding-assisting enzymes. Furthermore, it orchestrates cell cycle checkpoints by activating the ERAD machinery, which selectively targets unfolded/misfolded proteins for ubiquitin-proteasomal degradation, thereby alleviating proteotoxic stress and restoring ER homeostasis [44].

##### Regulation of lipid metabolism

ATF6 plays a pivotal role in maintaining lipid composition and homeostasis within the ER, which is essential for restoring ER function [45].

##### Balance between cell survival and apoptosis

Under mild stress conditions, ATF6 promotes cell survival by enhancing adaptive responses. However, under sustained stress, ATF6 may induce the expression of CHOP, which serves as a pro-apoptotic signal and ultimately leads to cell death [46].

## Role of ERS in DR

**Fig. 1:**
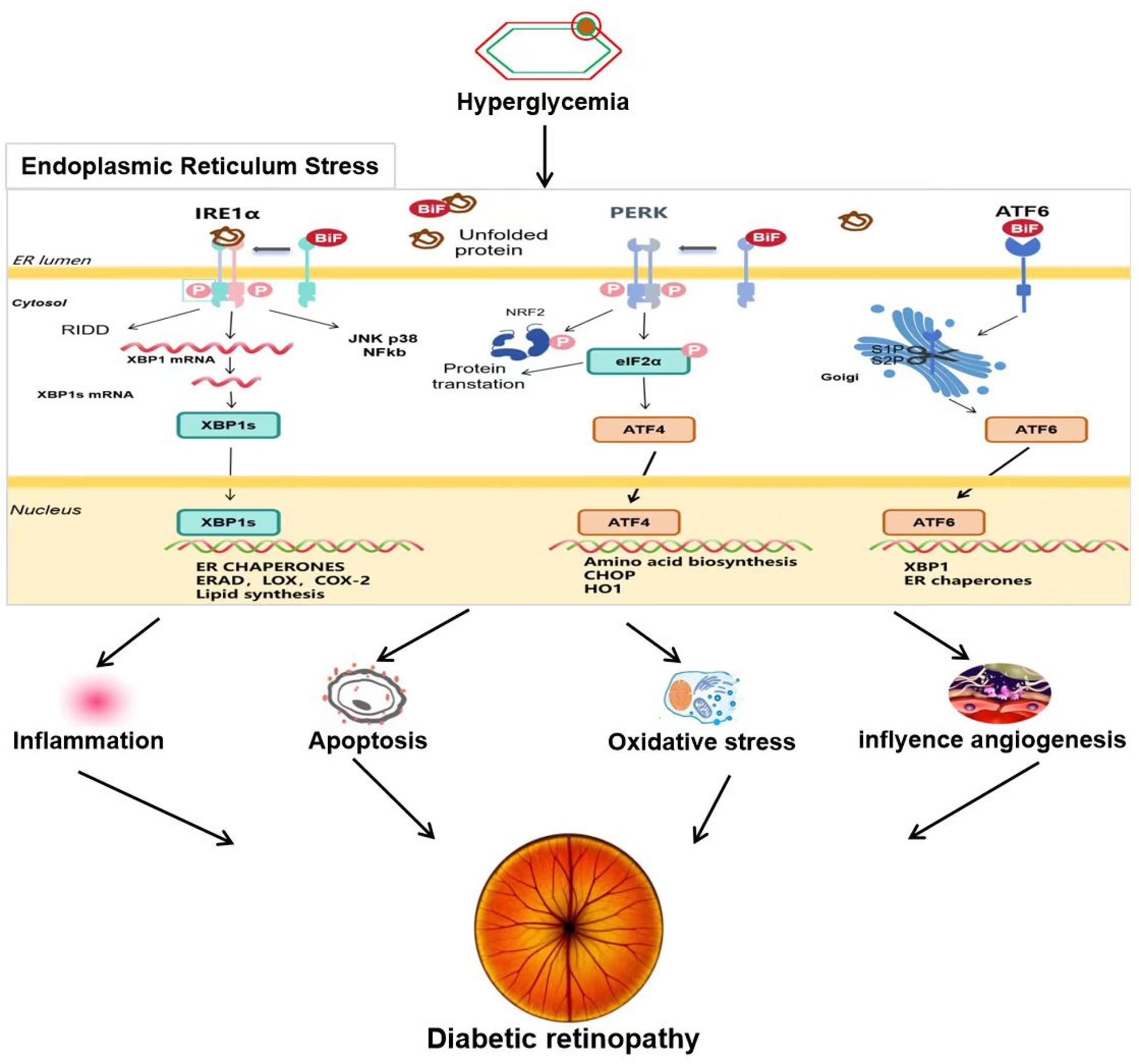
Schematic illustration of the role of endoplasmic reticulum stress (ERS) in the pathogenesis of diabetic retinopathy (DR). Under hyperglycemic conditions, oxidative stress and glucotoxicity lead to ER dysfunction and the accumulation of misfolded proteins, initiating ER stress. This activates the three principal UPR signaling branches: IRE1α pathway, which splices XBP1 mRNA to produce XBP1s, promoting the expression of ER chaperones and lipid synthesis but also activating JNK/p38/NF-κB to trigger inflammation and RIDD-mediated mRNA degradation; PERK pathway, which phosphorylates eIF2α to inhibit general protein translation while inducing ATF4, leading to the expression of genes involved in amino acid biosynthesis, antioxidant defense (e.g., HO-1), and pro-apoptotic regulators like CHOP; ATF6 pathway, which is activated upon translocation to the Golgi, where it is cleaved to its active form, enhancing transcription of ER chaperones and XBP1. Persistent or unresolved ER stress leads to chronic inflammation, apoptosis, oxidative stress, and pathological angiogenesis, all of which contribute to the progression of diabetic retinopathy

The role of ERS in DR has garnered increasing attention. Patients with diabetes are chronically exposed to hyperglycemia and oxidative stress, leading to impaired protein-folding function in the ER and triggering sustained activation of ERS [47]. This process significantly affects retinal structure and function through various mechanisms, including inflammation, oxidative stress, apoptosis, and abnormal angiogenesis. Eventually, it can promote the onset and progression of DR (Fig. 1) (Table 1).

**Table 1:** Key ERS targets and their direct roles in diabetic retinopathy (DR)

ERS component	Mechanism	DR pathological outcome	Intervention (Effect)
IRE1-XBP1S	Binds VEGF promoter	Neovascularization, BRB breakdown	STF-083010 (↓VEGF )
PERK-CHOP	Suppresses Bcl-2, activates Bax	Endothelial apoptosis, capillary dropout	GSK2606414 (↓Apoptosis )
ATF6	Upregulates GRP78/CHOP	Photoreceptor degeneration	ATF6 SiRNA (↑Cell survival )

↓: reduce, ↑:improve

This table summarizes the major ERS-related molecular components implicated in DR, including their pathological mechanisms, effects on retinal vascular and neuronal integrity, and corresponding pharmacological inhibitors that have shown therapeutic potential in preclinical models

### Promoting inflammation

ERS significantly exacerbates the inflammatory response in DR by activating NF-κB and NLRP3 inflammasome pathways [4, 48]. In a high-glucose environment, the IRE1 pathway plays an important role in the activation of ERS. Activated IRE1 binds to TNF receptor-associated factor 2 (TRAF2) via its endonuclease activity. Subsequently, this leads to activation of the IKK complex and facilitates the translocation of NF-κB from the cytoplasm to the nucleus [49]. The transcriptional regulation mediated by NF-κB induces the release of pro-inflammatory cytokines such as TNF-α, IL-1β, and IL-6, These mediators directly act on retinal endothelial cells, leading to dysregulation of tight junction protein expression and impairment of blood–retinal barrier integrity [50]. Additionally, these pro-inflammatory factors significantly increase vascular permeability and induce vascular leakage, thereby aggravating retinal edema and exudation [51]. Simultaneously, TNF-α can induce endothelial cell apoptosis, further exacerbating capillary occlusion and hypoperfusion, which constitute key early pathological changes in DR [52, 53].

Moreover, ERS amplifies the inflammatory response in the retina by promoting the activation of the NLRP3 inflammasome. The NLRP3 inflammasome mediates the maturation and secretion of IL-1β and IL-18 through caspase-1 [54]. These pro-inflammatory cytokines recruit immune cells, such as neutrophils and macrophages, into the retinal microcirculation, exacerbating endothelial cell damage and microvascular occlusion. IL-1β also induces thickening of the retinal capillary basement membrane and narrowing of the lumen, leading to local hypoxia, which further activates VEGF expression and promotes pathological neovascularization [48]. Overactivation of NLRP3 not only causes microvascular pathology but also exerts direct toxic effects on retinal ganglion cells via inflammatory mediators, inducing apoptosis and neurodegeneration [55]. Notably, the interaction between ERS and NLRP3 forms a robust positive feedback loop: ERS induced by the high-glucose environment continuously activates NLRP3, while the release of inflammatory factors and production of ROS further exacerbate ERS activation, maintaining the retina in a chronic inflammatory state [56].

Inflammation plays a dual role in DR: it leads to structural damage of the BRB and vascular leakage by directly destroying endothelial cells and tight junction proteins [57]; simultaneously, inflammatory factors induce infiltration of immune cells, releasing proteases and matrix metalloproteinases (MMPs), degrading the extracellular matrix and further destroying the capillary structure. The cascade effect of these inflammatory responses leads to typical retinal edema, exudation, microaneurysms, and vascular occlusion [58]. Through these mechanisms, ERS maintains and amplifies the inflammatory microenvironment, keeping retinal tissue in a chronic state of damage for a prolonged period.

Both experimental studies and clinical observations have confirmed the synergistic effect of ERS and inflammation in DR. Rat Müller cell lines stimulated with high glucose mimicked a diabetic environment in vitro. Data indicated that ERS was observed under high-glucose conditions. Activation of UPR signaling, including IRE1α–XBP1, PERK–ATF4, and ATF6 pathways, leads to increased expression of ER stress markers (GRP78, ATF4, XBP1s, and ATF6) as well as the angiogenic factor VEGF in experimental models of diabetic retinopathy. In contrast, genetic or pharmacological inhibition of these pathways significantly reduces the expression of these molecules. The three UPR-mediated inflammatory pathways are interdependent [59]. Inhibition of ER stress significantly reduced IL-1β and TNF-α secretion and improved retinal edema and vascular leakage [60]. Clinical reports have shown structural changes in different layers of the retina after metal exposure. Toxic metal exposure can cause retinal inflammation and damage through oxidative stress, apoptosis, DNA damage, and ERS [61]. However, the contribution of toxic metal exposure to retinal inflammation and damage remains controversial and appears to be context-dependent.

Although ERS-mediated inflammatory pathways have been widely recognized, the dynamic activation of different ERS signaling pathways and their cell-specific expression patterns during different stages of DR progression remain unclear. Future research should explore how ERS signaling pathways synergistically or competitively regulate NLRP3 and NF-κB pathways during the early versus advanced stages of DR [62]. Additionally, employing single-cell RNA sequencing techniques to investigate cell-specific effects of ERS signaling in the retina will provide critical data for developing more precise targeted therapies [63].

### Exacerbating oxidative stress

ERS is an important source of oxidative stress, and its role in DR has attracted increasing attention. Under high-glucose conditions, the activation of ERS significantly increases the production of intracellular ROS through the IRE1 and PERK pathways [64]. Specifically, the activation of IRE1 accelerates ROS generation through its downstream signaling molecule JNK [65], while the PERK pathway reduces the levels of antioxidant enzymes by affecting the expression of the antioxidant transcription factor nuclear factor erythroid 2-related factor 2 (Nrf2), further impairing the cell’s ability to clear ROS [66]. The continuous accumulation of unfolded proteins in the ER increases the load on protein folding and disrupts the functional coupling between the ER and mitochondria, resulting in calcium ion leakage from the ER to the mitochondria. This process not only enhances mitochondrial ROS generation but also leads to the loss of mitochondrial membrane potential, further aggravating cellular oxidative stress [67].

The role of oxidative stress in DR exhibits complex positive feedback characteristics. Overproduction of ROS disrupts the integrity of the BRB by directly damaging retinal endothelial cells and nerve cells [68]. Oxidative stress-induced lipid peroxidation leads to structural instability of the cell membrane, thereby increasing vascular permeability and leakage, and this loss of barrier function further results in retinal edema and microangiopathy [69]. Additionally, ROS induces programmed death of retinal nerve cells by activating apoptosis-related pathways, a process that is particularly dependent on the maintenance of mitochondrial membrane potential. Following the loss of mitochondrial membrane potential, mitochondria release cytochrome C, which subsequently activates the caspase cascade, ultimately resulting in extensive apoptosis of ganglion and endothelial cells [70]. The accumulation of ROS not only directly leads to cell damage but also further amplifies ERS activity by activating ER stress signaling pathways. For example, the IRE1 pathway can enhance the accumulation of unfolded proteins in the ER through JNK feedback, and the PERK pathway can promote the expression of pro-apoptotic genes via CHOP [71]. Thus, a vicious cycle between ERS and oxidative stress is formed.

It is worth noting that oxidative stress induced by ER stress exacerbates the disturbance of energy metabolism in retinal cells through mitochondrial dysfunction [72]. Under high-glucose conditions, retinal cells exhibit enhanced glycolytic flux and increased mitochondrial metabolic activity; however, mitochondrial function becomes inefficient due to oxidative stress, leading to excessive reactive oxygen species production and impaired ATP utilization rather than true energy deficiency [73]. Energy metabolism disorders not only prevent retinal cells from maintaining normal physiological functions but also make them more vulnerable to ROS damage [74]. Furthermore, oxidative stress reduces the cell’s ability to clear ROS by inhibiting antioxidant signaling pathways. For example, the key antioxidant transcription factor Nrf2 is inhibited by the PERK pathway under high-glucose conditions, and the expression of its downstream antioxidant genes, such as superoxide dismutase (SOD) and catalase (CAT), is significantly reduced. Dysregulation of this antioxidant defense mechanism accelerates ROS accumulation [75].

In DR, oxidative stress induces damage to retinal blood vessels and nerve cells, forming an interactive network of inflammation, apoptosis, and metabolic disorders [76]. ROS not only serves as an important mediator of inflammatory responses but also promotes the secretion of inflammatory factors by activating the NLRP3 inflammasome, exacerbating the retinal immune response [77]. The release of inflammatory factors such as TNF-α and IL-1β further increases ROS production and activates ER stress, forming a typical positive feedback loop [78, 79]. This complex interaction not only promotes the occurrence and development of DR but also renders its pathological process highly irreversible.

Experimental studies have demonstrated that ERS inhibitors, such as 4-phenylbutyric acid (4-PBA) and tauroursodeoxycholic acid (TUDCA), effectively reduce ROS production and improve retinal oxidative stress in animal models of DR [80, 81]. Furthermore, antioxidants such as N-acetylcysteine (NAC) significantly mitigate retinal cell damage by directly scavenging ROS [82]. In summary, ERS exacerbates oxidative stress through multiple mechanisms, playing a critical role in the pathological process of DR while also providing a potential therapeutic target for the development of novel treatment strategies.

Current research predominantly focuses on the IRE1-JNK and PERK-Nrf2 axes in ERS-mediated ROS generation, often overlooking the distinct responses of various retinal cell types under oxidative stress conditions. Future investigations should elucidate how ERS specifically regulates antioxidant responses in retinal microvascular endothelial cells, Müller cells, and ganglion cells, with particular emphasis on defining the selective therapeutic efficacy of ERS pathway inhibitors on these specific cell types [72]. Additionally, metabolomic studies exploring the interplay between ERS-mediated oxidative stress and metabolic disturbances in retinal cells may uncover new opportunities for metabolic-targeted therapies in DR [83].

### Induction of apoptosis

ERS induces apoptosis in retinal cells through various molecular pathways, representing a critical mechanism of cellular damage in DR. The PERK pathway plays a pivotal role in the sustained activation of ERS. Specifically, the PERK pathway significantly promotes apoptosis by upregulating the expression of the pro-apoptotic factor CHOP. CHOP downregulates the expression of anti-apoptotic factor Bcl-2 while upregulating the pro-apoptotic factor Bax, thereby disrupting mitochondrial membrane integrity, increasing mitochondrial outer membrane permeability, and releasing cytochrome C from mitochondria into the cytoplasm [84]. Released cytochrome C further activates caspase-9, initiating downstream apoptotic signaling cascades, including the activation of caspase-3, ultimately leading to programmed cell death [85]. In this process, the loss of mitochondrial membrane potential and reduced ATP production exacerbate energy metabolism disturbances in retinal cells, rendering them more susceptible to external damage [86]. Additionally, ERS further promotes apoptosis by inducing the activation of endoplasmic reticulum-specific apoptosis factor caspase-12 [87]. Caspase-12 directly acts on ERS-related signaling pathways, mediating cell death via caspase-3 activation, particularly in retinal endothelial cells and ganglion cells [88].

In the pathological progression of DR, ERS-induced apoptosis significantly disrupts the integrity of the BRB. Endothelial cell apoptosis leads to increased capillary permeability and structural disintegration, resulting in plasma protein and fluid leakage into retinal tissue, causing retinal edema and exudation [87]. Simultaneously, pericyte loss intensifies capillary occlusion, further reducing retinal blood perfusion efficiency, and leading to local tissue hypoxia and metabolic abnormalities [89]. These early pathological changes not only impair retinal vascular function but also promote abnormal neovascularization by increasing the expression of angiogenic factors such as VEGF [90]. The apoptosis of retinal nerve cells is a key driver of neurodegeneration in DR. High-glucose-induced ERS directly damages ganglion cells via CHOP and caspase-12 pathways, impairing visual signal transmission [91]. Studies have shown that ganglion cell loss occurs early in DR and serves as an important marker of decreased visual function [92]. Furthermore, retinal photoreceptor cells are also affected by ERS, with their apoptosis further exacerbating degenerative changes in retinal function [93].

ERS forms a positive feedback loop for apoptosis through a multi-layered mechanism. During retinal cell apoptosis, ERS is further activated; for example, the accumulation of unfolded proteins increases ER load, while mitochondrial membrane potential loss and ROS overproduction intensify ER-mitochondrial interactions, thereby aggravating ERS and cell death [94, 95]. This vicious cycle renders the pathological process of DR highly irreversible and accelerates disease progression. Experimental studies have demonstrated that ERS inhibitors effectively reduce CHOP and caspase-3 expression in high-glucose-induced diabetic models, significantly decreasing retinal cell apoptosis levels [96].

ERS-induced apoptosis not only destroys retinal structural integrity but also accelerates multidimensional lesion progression through its interaction with inflammation and oxidative stress. CHOP and caspase-12, as key molecules in ERS-induced apoptosis, provide potential therapeutic targets for DR treatment. In-depth studies of these molecules’ specific mechanisms of action across different cell types will aid in developing more precise and effective interventions. The core feature of ERS-induced apoptosis lies in its complex signaling network and multiple feedback mechanisms, necessitating further research to clarify how to effectively block these pathways, thereby slowing retinal cell death and DR progression.

While ERS-induced apoptosis mechanisms have been extensively studied, the temporal and spatial coordination between CHOP and caspase-12 in driving apoptosis across different retinal cell subpopulations remains unclear. Future studies should investigate apoptotic characteristics across various stages of DR, particularly how early-stage apoptosis can be predicted or intervened. Additionally, the role of ERS-mediated apoptotic bodies in intercellular communication within retinal cells has not been elucidated. Investigating ERS-induced apoptotic bodies in intercellular signaling may reveal novel strategies to halt disease progression [97].

### Influence angiogenesis

ERS significantly affects neovascularization in PDR by regulating VEGF expression. VEGF is a key regulator of neovascularization, co-regulated by local retinal hypoxia and ERS activation [98]. Under retinal hypoxic conditions, ERS promotes VEGF gene transcription and expression [90]. Simultaneously, ERS activates XBP1 signaling through the IRE1 pathway, further enhancing VEGF secretion levels [59]. Together, these mechanisms lead to persistent VEGF overexpression in the retinal microenvironment, directly promoting neovascularization. However, these newly formed vessels often exhibit structural abnormalities, such as disordered endothelial cells, incomplete lumens, and thin basement membranes, significantly reducing vessel mechanical strength and making them prone to rupture and bleeding. Abnormal new vessel expansion is accompanied by fibrotic hyperplasia, with excessive fibrous tissue eventually leading to retinal traction detachment, severely damaging patient vision [8].

ERS not only directly influences angiogenesis through VEGF upregulation but also disrupts normal angiogenesis processes by interfering with vascular endothelial cell function. In the ERS-activated state, the PERK pathway inhibits vascular endothelial cell migration and proliferation capacity through CHOP upregulation, while mitochondrial dysfunction and ATP deficiency further impair endothelial cell proliferation potential [99, 100]. Dysregulated ER-mitochondria coupling triggers excessive ROS generation, disrupting endothelial cell cytoskeletal architecture and degrading extracellular matrix components. This oxidative damage impairs cellular motility and weakens adhesion capacity, ultimately compromising the reestablishment of functional vascular networks [101]. Moreover, high VEGF levels fail to form normal blood vessels and instead cause disordered new vessel expansion through excessive signaling activation [102]. This abnormal angiogenesis is a hallmark pathological feature of PDR, characterized by intra- and preretinal neovascularization and subsequent recurrent bleeding and fibrotic progression [103].

There exists a significant positive feedback mechanism between ERS and angiogenesis. Retinal hypoxia-induced VEGF overexpression further increases ER protein load by enhancing ERS pathways and elevating the activity of ERS-related signaling molecules, such as XBP1 and ATF6, which further promote VEGF secretion at the transcriptional level [96]. Simultaneously, inflammatory responses and oxidative stress induced by ERS further destroy retinal vascular endothelial cell function by activating the NLRP3 inflammasome and ROS production, forming a vicious cycle by stimulating VEGF expression. This complex interaction causes local retinal neovascularization to lose its normal regulatory mechanism, leading to the irreversibility of retinopathy [104].

Studies have shown that ERS markers such as GRP78 and CHOP are significantly elevated in vitreous fluids and retinal tissues of DR patients and are closely correlated with VEGF levels, indicating a key role of ERS in neovascularization [105]. Experimental studies have further confirmed that ERS inhibitors, such as TUDCA, can significantly reduce VEGF expression and improve abnormal neovascularization [106]. Concurrently, anti-VEGF therapies (e.g., bevacizumab) have demonstrated significant efficacy by directly inhibiting VEGF activity, and reducing retinal neovascularization and fibrosis degrees [107]. However, targeting VEGF alone is insufficient to fully reverse the pathological changes in the retina. The combination of ERS inhibitors and anti-VEGF drugs may exert synergistic effects, potentially reducing neovascularization while preserving the function of retinal endothelial cells.

Currently, research on ERS in abnormal angiogenesis during PDR predominantly focuses on VEGF expression, with limited attention given to non-VEGF angiogenic factors, such as angiopoietin-like 4 (ANGPTL4) and platelet-derived growth factor (PDGF), as well as the regulatory mechanisms underlying vessel maturation. Therefore, future studies should explore how ERS regulates the expression of various angiogenic factors at different stages of angiogenesis. For instance, investigations could focus on elucidating the role of ERS in modulating factors like ANGPTL4 and PDGF to enhance understanding of the complexity of angiogenesis. Additionally, the involvement of ERS pathways in endothelial-pericyte interactions, particularly in maintaining the stability of newly formed blood vessels, merits further exploration. Unraveling the mechanisms underlying these interactions may provide novel strategies for promoting vascular stabilization and preventing fibrosis, offering promising avenues for the development of therapeutic approaches for related diseases [108].

## Therapeutic interventions targeting ERS

Multiple potential therapeutic targets for ERS in DR have been proposed. Inhibition of ERS, modulation of the UPR signaling pathways, and combination strategies involving anti-inflammatory and antioxidant therapies may effectively alleviate retinal microvascular and neural damage, providing new insights into DR treatment (Tables 2 and 3).

**Table 2:** Comparison of UPR pathways in DR

Pathway	Activation mechanism	Key functions	Role in DR
IRE1	Unfolded proteins →Dimerization →XBP1 splicing	Protein folding via ERAD→Inflammation (JNK/NF-KB)→Apoptosis (CHOP/Bax)	Drives inflammation, oxidative stress, and retinal endothelial cell apoptosis
PERK	GRP78 dissociation →elF2a phosphorylation	Translational arrest→ Antioxidant response (ATF4)→Apoptosis (CHOP)	Mediates chronic oxidative stress and apoptosis in retinal neurons
ATF6	Cleavage in Golgi →ATF6F nuclear translocation	Chaperone synthesis (GRP78)→Lipid metabolism →Apoptosis (CHOP)	Balances adaptive UPR and apoptosis; modulates angiogenesis

An overview of the activation mechanisms, biological functions, and contributions of the three major UPR signaling pathways—IRE1, PERK, and ATF6—in the context of diabetic retinopathy. These pathways influence inflammation, oxidative stress, and apoptosis in retinal cells under hyperglycemic stress

**Table 3:** Experimental therapeutic strategies targeting ERS in DR

Strategy	Agents	Mechanism	Outcome in models
ERS Inhibitors	4-PBA, TUDCA	Reduce unfolded Protein load; stabilize ER membrane	↓Retinal edema, vascular leakage, apoptosis
IRE1 Inhibition	STF-083010	Block XBP1 splicing	↓Inflammation, ROS
PERK Inhibition	GSK2606414	Suppress CHOP expression	↓Retinal cell apoptosis
Anti-lnflammatory/Antioxidant	MCC950(NLRP3 inhibitor), NAC	Inhibit NLRP3; Scavenge ROS	↓Retinal edema, oxidative damage

↓: reduce

A summary of pharmacological interventions targeting ER stress in DR models, including chemical chaperones (e.g., 4-PBA, TUDCA), pathway-specific inhibitors (e.g., STF-083010, GSK2606414), and antioxidant/anti-inflammatory compounds. Reported outcomes include reductions in inflammation, oxidative damage, and retinal cell apoptosis

### ERS inhibitors

ERS inhibitors reduce ERS-induced damage to the retina by suppressing ER stress activation and restoring cellular homeostasis. Below are two commonly used ERS inhibitors and their mechanisms of action:

#### 4-PBA

4-PBA is a chemical chaperone that promotes proper protein folding and reduces the accumulation of misfolded proteins, thereby alleviating ERS. 4-PBA binds to the hydrophobic regions of misfolded proteins, preventing their aggregation and subsequent activation of the PERK pathway [109]. Studies have demonstrated that in animal models of DR, 4-PBA significantly reduces the expression levels of inflammatory factors such as TNF-α and IL-6, alleviating retinal edema and vascular leakage. Additionally, 4-PBA blocks IRE1α-XBP1s splicing, reducing downstream activation of NF-κB and the NLRP3 inflammasome [110, 111]. Furthermore, 4-PBA inhibits the PERK-eIF2α-ATF4-CHOP axis, reducing retinal cell apoptosis and protecting retinal structure and function [101].

#### TUDCA

TUDCA, a bile acid derivative, stabilizes the endoplasmic reticulum membrane and enhances protein folding capacity. TUDCA reduces ERS-induced oxidative stress and inflammation by inhibiting the overactivation of UPR signaling pathways. It enhances sarcoplasmic/endoplasmic reticulum Ca²⁺ ATPase 2a (SERCA2a) activity, reducing ER Ca²⁺ leakage into mitochondria, rescuing mitochondrial membrane potential, and lowering ROS production in diabetic retinas [112]. In DR models, TUDCA has shown a potential to reduce retinal capillary occlusion, restore blood-retinal barrier integrity, and inhibit abnormal neovascularization [113]. TUDCA has demonstrated safety in Phase II/III trials for amyotrophic lateral sclerosis (ALS), showing neuroprotective effects via ERS reduction [114]. Although not yet tested in DR clinical trials, its ability to cross the blood-retinal barrier in preclinical models supports its potential repurposing for DR therapy.

### UPR signaling pathway regulation

Selective modulation of the three major UPR signaling pathways-IRE1, PERK, and ATF6-is an important strategy to mitigate retinal damage caused by ERS.

#### IRE1 path

IRE1α inhibitors, such as STF-083010, block the endonuclease activity of IRE1α, preventing XBP1 mRNA splicing and subsequent activation of pro-inflammatory and angiogenic genes. In oxygen-induced retinopathy models, STF-083010 reduced retinal VEGF levels and abnormal neovascularization by disrupting XBP1s binding to the VEGF promoter [115]. Human retinal endothelial cells exposed to high glucose showed a 70% reduction in IL-6 secretion after IRE1α inhibition [59].

#### PERK path

The PERK pathway regulates protein translation and balance through eIF2α phosphorylation. When ERS is chronically activated, the PERK pathway may trigger pro-apoptotic signaling. PERK inhibitors, such as GSK2606414, prevent eIF2α phosphorylation, halting ATF4/CHOP-mediated apoptosis and oxidative stress. In streptozotocin (STZ)-induced diabetic mice, GSK2606414 decreased CHOP expression, reducing retinal ganglion cell apoptosis and preserving visual function [100]. PERK inhibition restored Nrf2 activity, increasing SOD and CAT levels in diabetic retinas [116].

#### ATF6 path

ATF6 acts as a sensor of ERS and alleviates stress responses primarily by activating molecular chaperones and folding enzyme genes. However, overactivation of ATF6 may be associated with abnormal inflammation and angiogenesis. Small molecule drugs targeting ATF6 inhibit the inflammatory response in retinal endothelial cells under high-glucose conditions while reducing the formation of abnormal new blood vessels [117].

### Anti-inflammatory and antioxidant strategies

ERS in DR often interacts with inflammatory responses and oxidative stress; therefore, combining anti-inflammatory and antioxidant strategies may effectively reduce the pathological effects of ERS.

#### Anti-inflammatory drugs

Anti-inflammatory drugs relieve inflammation in the retinal microenvironment by inhibiting inflammatory pathways and cytokine release. For example, TNF-α inhibitors such as adalimumab have been shown in animal models to reduce inflammatory responses and improve retinal function [118]. Additionally, NLRP3 inflammasome inhibitors such as MCC950 have been shown to reduce inflammation levels in DR and decrease retinal edema by blocking NLRP3 oligomerization and suppressing caspase-1-dependent maturation of IL-1β/IL-18 [119]. Treatment with MCC950 reduced NLRP3 activation in human retinal pigment epithelial cells exposed to a high-glucose environment [77].

#### Antioxidants

Antioxidants protect retinal cells from oxidative stress damage by reducing ROS production or scavenging excess reactive oxygen species. For example, NAC, a commonly used antioxidant, reduces ROS levels by enhancing glutathione synthesis. Diabetic mice treated with NAC exhibited lower retinal ROS, increased retinal ganglion cell survival, and preserved electroretinogram responses [120]. In vitro, NAC restored mitochondrial membrane potential in photoreceptors exposed to high glucose, reducing apoptosis [82].

### Gene editing technologies

Gene editing tools, particularly CRISPR-Cas9, offer unprecedented opportunities for precise modulation of ERS-related molecular pathways. Applying CRISPR-Cas9 in retinal regeneration studies using damaged zebrafish retinal models will undoubtedly enhance our understanding of the mechanisms underlying retinal repair and vision restoration in zebrafish [121].

In conclusion, ER stress and its associated pathological changes in DR can be effectively alleviated through ERS inhibitors, UPR signaling pathway modulation, and combined anti-inflammatory and antioxidant therapies [122, 123]. Current studies are predominantly based on preclinical models and require advancement into clinical trials of 4-PBA/TUDCA in DR, as well as exploration of their use in combination with anti-VEGF therapy or gene editing techniques [59].

## Outlook

Despite significant advancements in understanding ERS’s role in DR, several critical areas require further exploration. One pressing issue is the detailed analysis of spatiotemporal variations in ERS activation across different retinal cell populations, including endothelial cells, pericytes, Müller cells, and ganglion cells, throughout the progression of DR. Advanced scRNA-seq and spatial transcriptomics can offer invaluable insights by revealing cell-specific ERS pathways activation patterns, thus facilitating more targeted therapeutic strategies [63, 124].

Moreover, the dynamic interactions among the three UPR signaling branches-IRE1, PERK, and ATF6-remain incompletely understood across DR’s developmental stages. Future research should adopt real-time quantitative PCR, Western blotting, and single-cell analyses to precisely track and characterize pathway-specific activation shifts. These studies could identify critical regulatory nodes and potential therapeutic targets at various stages of DR progression, which could lead to stage-specific interventions and improve patient outcomes [59, 100, 117].

Beyond VEGF, the regulatory roles and mechanisms of non-classical angiogenic factors such as ANGPTL4 and PDGF in ERS-induced pathological angiogenesis have been inadequately investigated. Future studies should employ gene-editing tools such as CRISPR-Cas9 to selectively manipulate these genes’ expression in vitro and in vivo, thus providing a clearer understanding of how ERS modulates various angiogenic factors within the retinal microenvironment [108, 125].

Additionally, exploring the crosstalk between ERS-induced inflammation and oxidative stress pathways is vital for understanding the multifaceted pathology of DR. Integrating metabolomics and proteomics approaches can uncover novel interactions between metabolic dysfunction and ERS pathways, potentially revealing new metabolic targets for therapeutic intervention [83]. Furthermore, combination therapies involving ERS inhibitors (like 4-PBA and TUDCA), antioxidants (such as NAC), and anti-inflammatory agents (e.g., MCC950) require rigorous preclinical testing to evaluate their synergistic effects and translational potential [110, 113, 119].

Finally, to advance the clinical translation of ERS-targeting therapies, we propose the initiation of multicenter randomized controlled trials focusing on agents such as 4-PBA and TUDCA in patients with NPDR and PDR. Furthermore, the establishment of a biomarker-based patient stratification system incorporating ERS-related genes (e.g., CHOP, XBP1s) and circulating cytokine profiles could support precision treatment strategies. Integrating such approaches will not only validate the therapeutic potential of ERS modulators but also provide a practical roadmap for their clinical implementation [114].

## Summary

This review comprehensively explores the intricate roles of ERS in the pathogenesis of DR, emphasizing the molecular mechanisms underlying ERS and its interaction with inflammation, oxidative stress, apoptosis, and pathological angiogenesis. The critical involvement of the three primary UPR signaling pathways (IRE1, PERK, and ATF6) in modulating these processes underscores the complexity of DR pathology. Furthermore, various therapeutic approaches targeting ERS, including chemical chaperones (4-PBA, TUDCA), UPR pathway-specific modulators (STF-083010, GSK2606414), antioxidants (NAC), and anti-inflammatory agents (adalimumab, MCC950), demonstrate potential efficacy in preclinical models. However, significant research gaps remain, necessitating future studies to enhance the mechanistic understanding and facilitate the translation of these promising therapeutic strategies into clinical practice, ultimately aiming to preserve vision and improve the quality of life for diabetic patients.

## Data Availability

Data sharing does not apply to this article as no new data were created or analyzed in this study.
